# The Order on Parasitic Mange

**Published:** 1918-09-21

**Authors:** 


### The Order on Parasitic Mange.

The Board of Agriculture has caused to be issued Leaflet No. 8, with an attachment of a set of rules to be followed for the prevention and cure of mange in horses. The disease, though not unknown before, appears to have become more common in the last few years, though there does not appear to be any obvious connection between it and the war. Though not actually dangerous to life nor of a nature to incapacitate a horse from work, yet the disease greatly reduces a horse's efficiency by disturbing its rest and by the actual loss of strength caused by the feeding of thousands of parasites.

The disease takes two forms—the sarcoptic, the more troublesome because the more difficult to extirpate, and the psoroptic. It is, however, not in the competency of the ordinary horse-owner to distinguish the one form from the other. That is the function of the veterinary surgeon. It is the duty of every horse-owner, and default involves penalties of fine or imprisonment, to notify to the police any suspicious case. Suspicion should always be roused by constant rubbing and other evidence of itchiness. An itchy horse will often rub itself raw, especially about the tail and the neck. It will also show the greatest pleasure on being scratched, leaning towards the scratcher just as a pig will. The disease is very infectious and very troublesome to get rid of. All horse-owners will be wise to apply to the Secretary, the Joint Committee of the Board of Agriculture and Ministry of Food, 6a Dean's Yard, Westminster, for a free and post-free copy of Leaflet No. 8 and the rules.

